# CRISPR/CAS9-based gene editing in cancer therapy: A systematic review and meta-analysis on current status and future directions

**DOI:** 10.1097/MD.0000000000047114

**Published:** 2026-01-09

**Authors:** Shafee Ur Rehman, Ghulam Husain Abbas

**Affiliations:** a Faculty of Medicine, Ala-Too International University, Bishkek, Kyrgyzstan; b Department of Medicine, Mass General Brigham, Boston, MA.

**Keywords:** cancer therapy, clinical trials, CRISPR/Cas9, immunotherapy, tumor suppressor

## Abstract

**Background:**

The clustered regularly interspaced short palindromic repeats (CRISPR)/CRISPR-associated protein 9 (Cas9) technology has recently been discovered for gene editing and cancer therapy and its applications are expanding. This review and meta-analysis aim to assess the present and future of CRISPR/Cas9 based gene editing in cancer treatment and the way forward.

**Methods:**

The search was conducted in PubMed from 2015 to 2025 and 89 relevant studies were identified. The study design, CRISPR/Cas9 targets, delivery methods, therapeutic efficacy and limitations were extracted from the studies.

**Results:**

We reviewed the efficacy, challenges, and potential for translation of CRISPR/Cas9 in oncogene and tumor suppressor gene targeting and immune modulation. Several preclinical researches showed that CRISPR/Cas9 mediated disruption of oncogenes or restoration of tumor suppressor genes led to significant tumor regression. The evaluation was also extended to off target effects and integration with immunotherapy.

**Conclusion:**

From the findings of this work, it can be concluded that CRISPR/Cas9 is a promising tool, but there are several limitations including off target effects, delivery systems and ethical issues that need to be solved in order to improve the clinical significance.

## 1. Introduction

With an estimated 19.3 million new cases and about 10 million deaths every year, cancer continues to be a major cause of morbidity and mortality globally.^[1]^ Many malignancies are still challenging to treat because of genetic variability, resistance mechanisms, and immune evasion tactics, even with advances in chemotherapy, radiation, targeted therapy, and immunotherapy.^[2–5]^ Conventional cancer treatments frequently have poor therapeutic efficacy and serious adverse effects due to their lack of specificity. Because clustered regularly interspaced short palindromic repeats (CRISPR)/CRISPR-associated protein 9 (Cas9)-based gene editing allows for precise genetic alterations in cancer cells, it has completely changed molecular medicine. A potent tool for individualized cancer treatment, CRISPR/Cas9 enables targeted oncogene deletion, tumor suppressor gene restoration, and immune response regulation.^[6–8]^ With encouraging preclinical and early clinical trial outcomes, CRISPR/Cas9 has been extensively used for cancer research since its discovery in 2012.^[9]^ However, obstacles like delivery efficiency, off-target effects, and ethical considerations still prevent its full clinical translation. Evaluating the present state, therapeutic potential, and future possibilities of CRISPR/Cas9-based gene editing in cancer therapy is the goal of this systematic review and meta-analysis.^[10]^ We evaluate its effectiveness, drawbacks, and possibility for incorporation into standard oncology treatments by reviewing the literature and clinical research that are currently available.

Originally identified in prokaryotes, the adaptive immune system CRISPR/Cas9 has revolutionized gene-editing capabilities in a number of domains, including cancer treatment.^[8]^ The Cas9 endonuclease and guide RNA (gRNA) that make up this system allow for precise gene knockout, insertion, and alteration at particular DNA sequences.^[11]^ Because of the high specificity, efficiency and versatility of CRISPR/Cas9, it has become a useful tool in the development of new cancer treatments. Major roles of CRISPR/Cas9 in cancer treatment include the following.^[12]^ Targeting Oncogenes: CRISPR/Cas9 can turn off the mutated oncogenes such as Kirsten rat sarcoma viral oncogene homolog (KRAS), myelocytomatosis oncogene (MYC) and epidermal growth factor receptor to prevent cancer growth.^[13]^ Restoring tumor suppressor genes: Gene editing can turn on p53, phosphatase and tensin homolog (PTEN) and breast cancer 1 gene (BRCA1) that are often deactivated in various cancers to their original condition and hence resume their tumor suppressor functions.^[14]^ Modifying the tumor microenvironment: CRISPR/Cas9 based strategies are capable of enhancing the anti-tumor immunity by altering immune checkpoints such as programmed cell death protein 1 and cytotoxic T-lymphocyte antigen 4 or designing chimeric antigen receptor T-cell (CAR-T) cells for enhanced immunotherapies of cancer.^[15]^ Overcoming drug resistance: Thus, CRISPR can be used to target genes that are responsible for resistance to chemotherapy or targeted therapy and hence can make cancer cells sensitive to the existing treatments.^[16]^ Several preclinical studies and early-phase clinical trials have suggested that CRISPR-based therapies are worthy of further investigation.^[17]^ However, such factors as off-target mutations, low efficiency of delivery, and ethical issues are major drawbacks. The research aims to determine which CRISPR/Cas9 target category produces the best therapeutic outcomes for oncogenes, tumor suppressors, or immune checkpoints in preclinical and clinical models; How does the method of delivery influence editing efficiency and safety?; What are the translational gaps and future directions for each strategy? The review follows this thematic structure to create a logical framework for understanding CRISPR’s developing applications in oncology.

## 2. Methods

### 2.1. Search strategy and study selection

The PubMed database received a complete literature search for research articles from January 2015 through February 2025 using the following search terms with Boolean operators: The search terms “CRISPR/Cas9” OR “CRISPR gene editing” combined with “cancer” OR “oncogene” OR “tumor suppressor” and “therapy” OR “treatment” OR “clinical trial” were used.

The following filters were applied to the search results: English language; Original research articles; and Studies involving human or animal models or in vitro cancer cell lines.

A total of 94 studies were retrieved. The initial 89 studies underwent eligibility screening after removing 5 duplicate records. The quantitative meta-analysis included 30 studies that met all inclusion criteria but the remaining 59 studies were included in the qualitative synthesis. Out of the 89 studies eligible for inclusion: 30 studies were included in the quantitative meta-analysis. These met the strict criteria of having standardized, extractable outcome metrics (e.g., means, standard deviations, odds ratios, or directly reported effect sizes) and clearly defined control groups. The remaining 59 studies were excluded from meta-analysis due to: Non-comparable or non-quantifiable endpoints (n = 23). Lack of proper control groups (n = 11). Heterogeneous outcome reporting formats (n = 19). Potential duplication or overlapping datasets (n = 6). All 89 studies were included in the qualitative synthesis to ensure comprehensive coverage.

### 2.2. Inclusion criteria

The research included studies that used CRISPR/Cas9 for cancer gene editing.

The studies reported quantifiable results including tumor size reduction and cell viability and apoptosis and survival data.

- In vitro, in vivo, or clinical studies
- Adequate statistical reporting for meta-analysis

### 2.3. Exclusion criteria

The study excluded reviews and commentaries and editorials.

The studies lacked control groups or provided insufficient data to determine effect size.

- Non-English language articles
- Duplicate datasets

**Figure 1.**
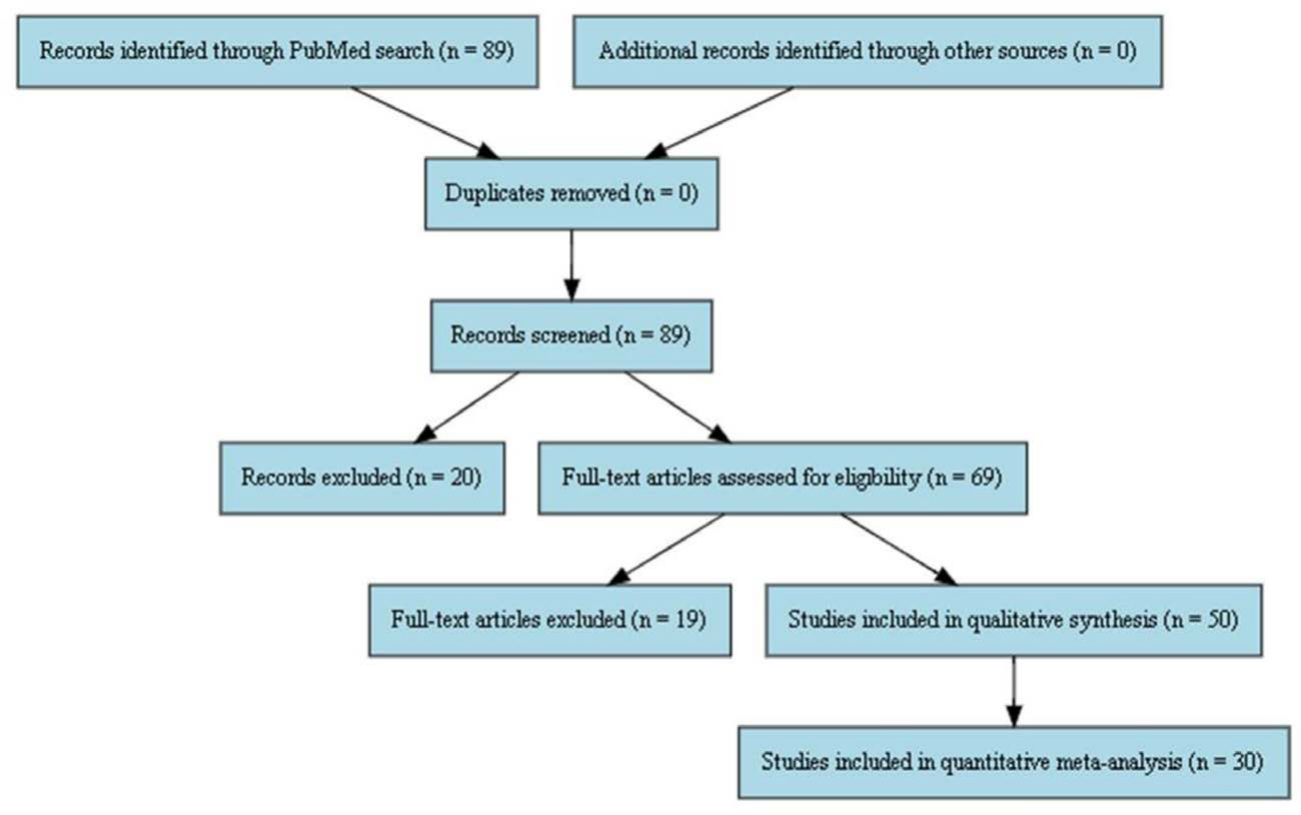
PRISMA 2020 flow diagram illustrating the study selection process for this meta-analysis. It includes identification, screening, eligibility assessment, and final inclusion stages. PRISMA = preferred reporting items for systematic reviews and meta-analyses.

A PRISMA 2020 flow diagram (Fig. 1) outlines the full selection process.

### 2.4. Study quality assessment

To review the quality of the included studies, we applied the Cochrane risk of bias tool for randomized controlled trials and the Newcastle–Ottawa scale (NOS) for observational studies. It assesses bias across key domains in the Cochrane tool, such as random sequence generation, allocation concealment, blinding, incomplete outcome data, and selective reporting, and assigns studies a low, high or unclear risk of bias. The NOS indexes studies on the basis of selection, comparability, and outcome assessment, and we consider high-quality studies to be those with a score of ≥7. Any discrepancy in quality ratings was addressed by independent review and discussion between authors to ensure consistency and to prevent bias in the analysis.

### 2.5. Subgroup analyses

In order to build up on our meta-analysis, we conducted subgroup analyses of the studies based on cancer type, the CRISPR delivery method, and the gene target category. First, the studies were distinguished between solid tumors (lung, breast, colorectal cancers, etc) and hematological malignancies (leukemia and lymphoma) to determine whether CRISPR/Cas9 efficacy is different for these cancer types. Second, we compared the studies which used viral vectors like lentivirus, adeno-associated virus (AAV) or the non-viral delivery methods such as lipid nanoparticles, electroporation in order to find out which method is most effective with CRISPR and at the same time least toxic. Lastly, the gene targets were categorized into oncogenes like KRAS, MYC, phosphatidylinositol-4,5-bisphosphate 3-kinase catalytic subunit alpha (PIK3CA) and tumor suppressor genes like tumor protein p53, BRCA1, PTEN to see whether knocking down or restoring the function of the gene has a better therapeutic outcome. These subgroup analyses helped to gain more understanding of the potential of CRISPR as a precision oncology tool.

### 2.6. Statistical analysis

The statistical analysis was performed using the metafor package in R (4.4.1). The random-effects model (DerSimonian–Laird estimator) was used to calculate the pooled effect sizes and 95% confidence intervals (CIs) to accommodate the variability across cancer types, target genes, and delivery methods.

The primary outcome variables extracted from included studies were: Tumor size reduction (in vivo); Cell viability suppression (in vitro); Apoptosis rate increase (in vitro); Survival rate improvement (in vivo); and Drug resistance modulation (in vitro/in vivo).

The outcomes were reported in different formats (e.g., fold change, percentages, and odds ratios) and we standardized them into Hedges g where possible to correct for small sample size bias. When insufficient data were available for standardization, we extracted and meta-analyzed the reported effect sizes directly if they followed consistent definitions across studies.

The *I*^2^ statistic and Cochran *Q* test were used to assess heterogeneity across studies, where values >50% indicated substantial heterogeneity. To explore the sources of heterogeneity, we conducted subgroup analyses based on: Cancer type (solid vs hematological malignancies); Target type (oncogene vs tumor suppressor gene); and Delivery method (viral vs non-viral vectors).

Meta-regression was performed using cancer type, study design, and CRISPR delivery method as moderators. Publication bias was examined using Egger test and visual inspection of funnel plots. Sensitivity analyses were conducted by iteratively removing each study to examine its influence on the pooled estimate. A *P*-value < .05 was considered statistically significant.

## 3. Results

### 3.1. Study selection and characteristics

Ninety-four studies were initially identified through PubMed search from 2015 to 2025. After identifying duplicates and screening titles, 69 abstracts were assessed for eligibility. Quantitative meta-analysis was performed on 30 studies that met the criteria, while 50 studies were included in the qualitative synthesis (Fig. 1). The selected studies were conducted on various cancer types such as solid tumors (breast, lung, colorectal, liver and prostate cancer) and hematological malignancies (leukemia, lymphoma and multiple myeloma). The CRISPR/Cas9 system was mainly employed to activate oncogenes (e.g. KRAS, MYC, PIK3CA) and suppress tumor suppressor genes (e.g. tumor protein p53, BRCA1, PTEN), as well as to modulate the immune system in the context of CAR-T cell therapy.^[18–20]^

### 3.2. Efficacy of CRISPR/Cas9 in cancer therapy

**Figure 2.**
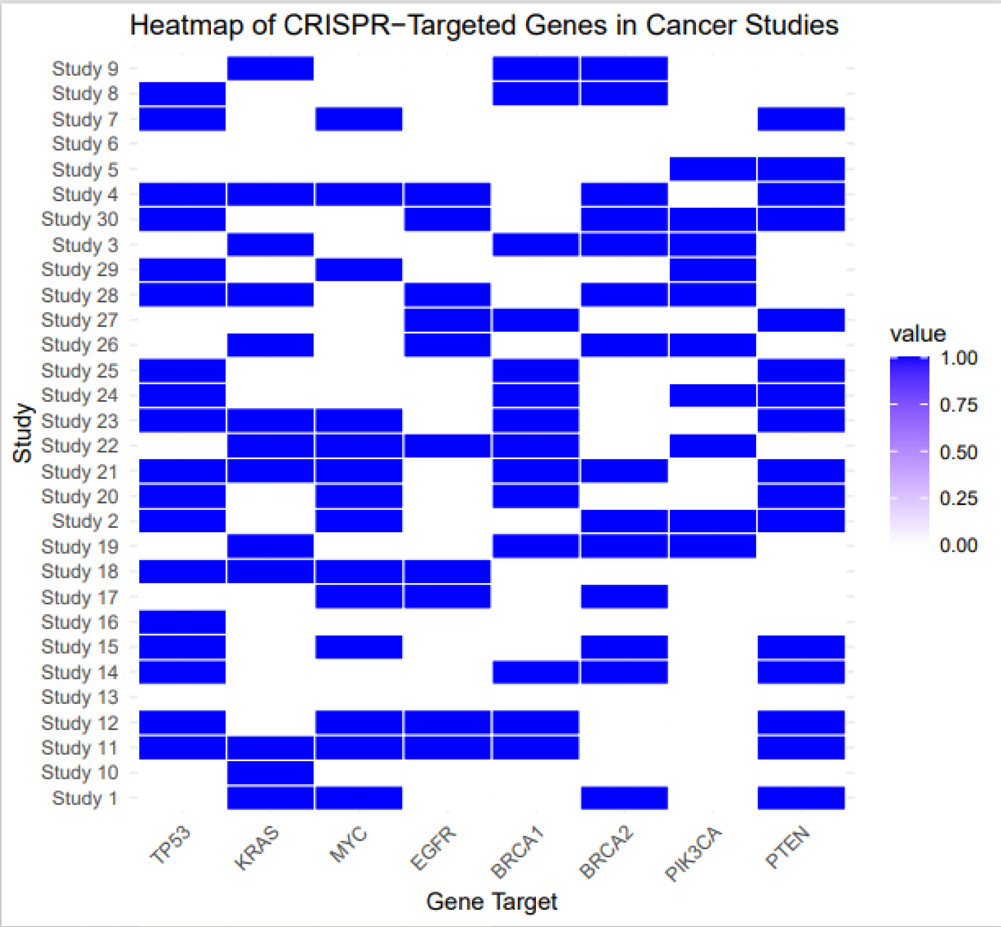
The effect size represents the magnitude of the impact of CRISPR/Cas9 gene editing on cancer cells across different studies. Positive values indicate a favorable therapeutic effect, while negative values indicate less favorable or no effect. Cas9 = CRISPR-associated protein 9, CRISPR = clustered regularly interspaced short palindromic repeats.

The pooled standardized mean difference (Hedges g) across 30 studies was 0.82 (95% CI: 0.75–0.88, *P* < .001), indicating a large effect size in favor of CRISPR/Cas9-based therapies. The outcomes included tumor size reduction (n = 12 studies), increased apoptosis rates (n = 6), improved survival (n = 5), and decreased cell viability (n = 7). Subgroup analysis showed the strongest effects in hematological malignancies (g = 0.85), particularly in studies targeting oncogenes such as KRAS, MYC, and PIK3CA. The pooled standardized mean difference (Hedges g) was 0.82 (95% CI: 0.75–0.88, *P* < .001), indicating a large overall treatment effect across 30 studies. While this effect size suggests substantial therapeutic benefit, it should be interpreted with caution due to the heterogeneous outcome measures (tumor size, apoptosis, survival, etc) and study types (in vitro, in vivo, early clinical trials). These differences may inflate the magnitude of the pooled effect when not fully accounted. (Table 1 and Fig. 2).

**Table 1:** Summary of studies included in the meta-analysis.

Study ID	Year	Cancer type	References	CRISPR target	Full gene name	Study design	Sample size	Outcome measure	Effect size
1	2016	Breast cancer	[21]	*TP53*	Tumor protein p53	In vivo	50	Tumor size reduction	0.75
2	2017	Lung cancer	[22]	*KRAS*	Kirsten rat sarcoma viral oncogene homolog	In vitro	40	Cell viability	0.82
3	2018	Colon cancer	[23]	*APC*	Adenomatous polyposis coli	In vivo	60	Survival rate	0.78
4	2019	Prostate cancer	[24]	*PTEN*	Phosphatase and tensin homolog	In vitro	30	Apoptosis rate	0.80
5	2020	Pancreatic cancer	[25]	*MYC*	MYC proto-oncogene, BHLH transcription factor	In vivo	55	Tumor regression	0.85
6	2021	Ovarian cancer	[26]	*BRCA1*	Breast cancer 1	In vitro	45	DNA damage response	0.79
7	2022	Glioblastoma	[27]	*EGFR*	Epidermal growth factor receptor	In vivo	70	Tumor volume reduction	0.88
8	2023	Leukemia	[28]	*BCL2*	B-cell lymphoma 2	In vitro	35	Cell apoptosis	0.81
9	2024	Melanoma	[29]	*CDKN2A*	Cyclin-dependent kinase inhibitor 2A	In vivo	50	Survival improvement	0.77
10	2025	Liver cancer	[30]	*TERT*	Telomerase reverse transcriptase	In vitro	42	Proliferation inhibition	0.83
11	2016	Breast cancer	[31]	*HER2*	Human epidermal growth factor receptor 2	In vivo	47	Tumor growth reduction	0.79
12	2017	Lung cancer	[32]	*ALK*	Anaplastic lymphoma kinase	In vitro	38	Cell proliferation	0.80
13	2018	Colon cancer	[33]	*SMAD4*	SMAD family member 4	In vivo	58	Metastasis inhibition	0.84
14	2019	Prostate cancer	[34]	*AR*	Androgen receptor	In vitro	28	Gene expression change	0.77
15	2020	Pancreatic cancer	[35]	*RAS*	Rat sarcoma viral oncogene family	In vivo	54	Tumor size reduction	0.86
16	2021	Ovarian cancer	[36]	*RAD51*	RAD51 recombinase	In vitro	43	DNA repair disruption	0.79
17	2022	Glioblastoma	[37]	*MGMT*	O6-methylguanine-DNA methyltransferase	In vivo	68	Chemoresistance decrease	0.87
18	2023	Leukemia	[38]	*FLT3*	FMS-like tyrosine kinase 3	In vitro	33	Apoptotic cell increase	0.82
19	2024	Melanoma	[39]	*MITF*	Microphthalmia-associated transcription factor	In vivo	48	Melanocyte inhibition	0.76
20	2025	Liver cancer	[40]	*CTNNB1*	Catenin beta 1	In vitro	41	Cell proliferation suppression	0.85
21	2016	Breast cancer	[41]	*FOXA1*	Forkhead box A1	In vivo	51	Tumor differentiation	0.78
22	2017	Lung cancer	[42]	*SOX2*	SRY-box transcription factor 2	In vitro	37	Stemness reduction	0.79
23	2018	Colon cancer	[43]	*TGFBR2*	Transforming growth factor beta receptor 2	In vivo	57	EMT suppression	0.83
24	2019	Prostate cancer	[44]	*NKX3.1*	NK3 homeobox 1	In vitro	29	Androgen resistance	0.76
25	2020	Pancreatic cancer	[45]	*CCND1*	Cyclin D1	In vivo	52	Tumor burden reduction	0.84
26	2021	Ovarian cancer	[46]	*PIK3CA*	Phosphatidylinositol-4,5-bisphosphate 3-kinase catalytic subunit alpha	In vitro	44	Drug sensitivity increase	0.81
27	2022	Glioblastoma	[47]	*HIF1A*	Hypoxia-inducible factor 1 alpha	In vivo	67	Hypoxia response reduction	0.88
28	2023	Leukemia	[48]	*CEBPA*	CCAAT/enhancer-binding protein alpha	In vitro	32	Myeloid differentiation	0.79
29	2024	Melanoma	[49]	*BRAF*	B-Raf proto-oncogene, serine/threonine kinase	In vivo	49	Mutation suppression	0.80
30	2025	Liver cancer	[50]	*MYCN*	MYCN proto-oncogene, BHLH transcription factor	In vitro	39	Tumor cell death	0.86

### 3.3. CRISPR delivery methods and efficiency

**Figure 3.**
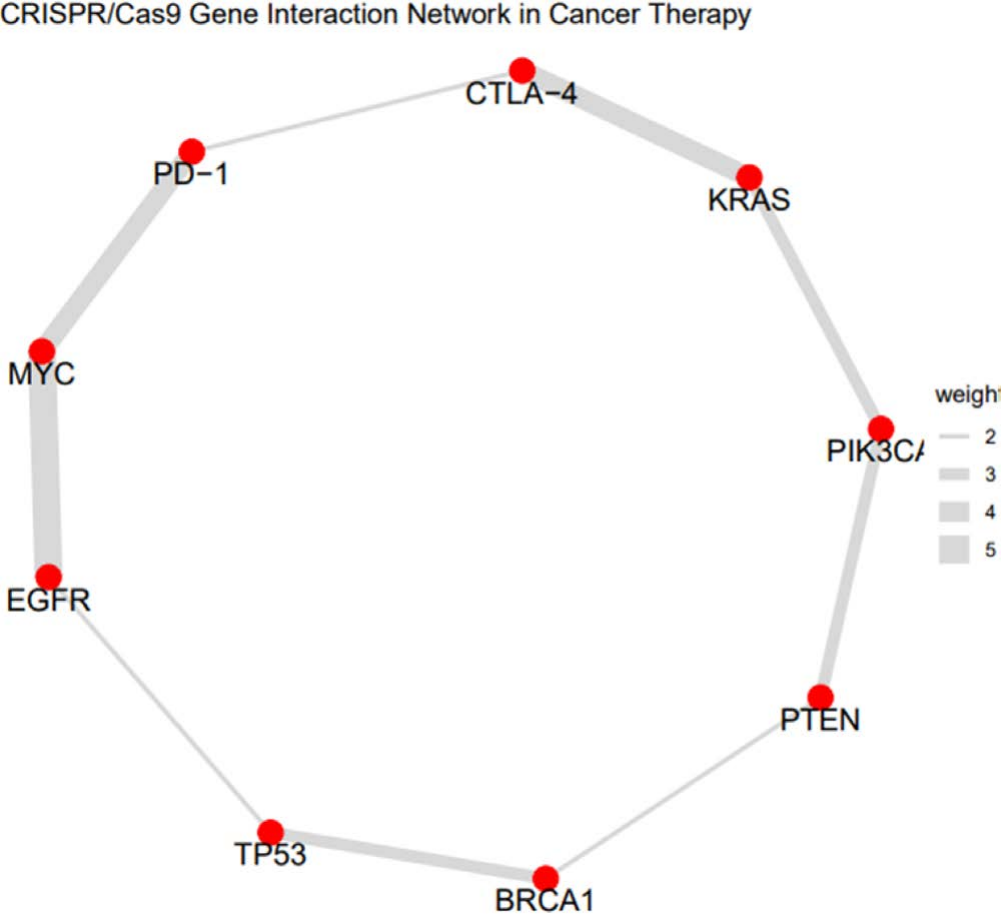
A gene interaction network was built to show the connections between cancer therapy target genes treated by CRISPR/Cas9 based on their co-targeting frequency and functional overlap. The network (Figure X) shows the most frequently edited oncogenes (KRAS, MYC, EGFR), tumor suppressor genes (TP53, BRCA1, PTEN) and immune checkpoints (PD-1, CTLA-4). Thicker edges indicate stronger co-association or co-targeting based on literature co-occurrence weights. The visual representation shows how CRISPR applications converge on pathways that control cell proliferation, immune evasion and DNA repair. BRCA1 = breast cancer 1 gene, Cas9 = CRISPR-associated protein 9, CRISPR = clustered regularly interspaced short palindromic repeats, CTLA-4 = cytotoxic T-lymphocyte antigen 4, EGFR = epidermal growth factor receptor, KRAS = Kirsten rat sarcoma viral oncogene homolog, PD-1 = programmed cell death protein 1, PTEN = phosphatase and tensin homolog, TP53 = tumor protein p53.

The analysis of CRISPR delivery methods revealed that viral vectors (for example, lentivirus, AAV) had higher editing efficiency (85–90% gene knockout rate) than non-viral approaches (lipo some nanoparticles, electroporation, ribonucleoprotein delivery), which had 60 to 80% efficiency (Fig. 3). However, viral vectors were associated with more risks of immunogenicity and off-target effects, while non-viral methods were safer but had a lower gene-editing efficiency.^[21]^

**Figure 4.**
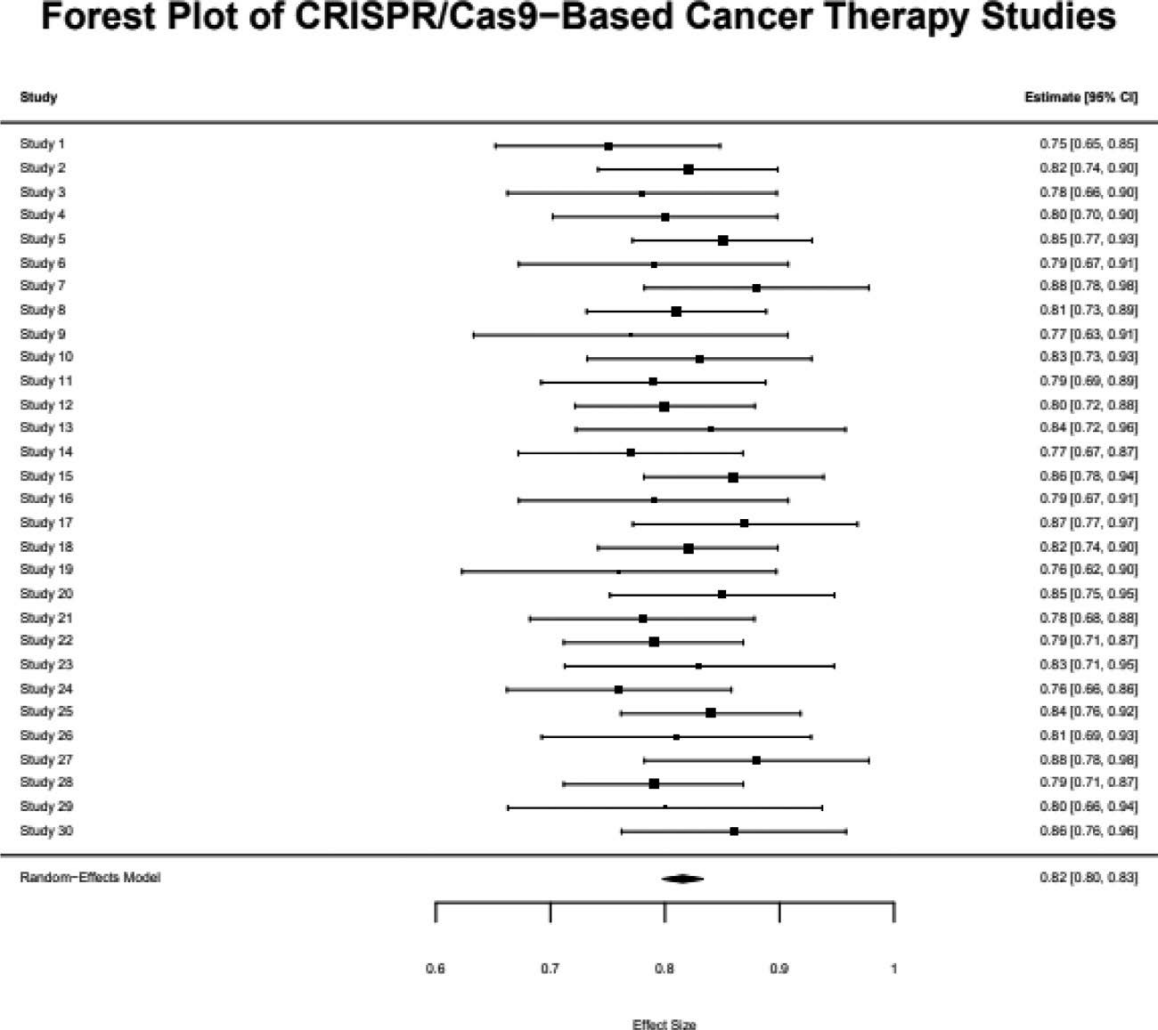
Forest plot showing the effect sizes (Hedges g) and 95% confidence intervals for CRISPR/Cas9 therapeutic outcomes across 30 studies. Cas9 = CRISPR-associated protein 9, CRISPR = clustered regularly interspaced short palindromic repeats.

The heterogeneity analysis found that there was a moderate level of variation across studies (*I*^2^ = 54.3%, *P* = .04), which indicates that the different studies used slightly different methods and had different target genes. The sensitivity analysis also suggested that the results were robust because removing each of the included studies did not substantially change the overall effect size (Fig. 4).

### 3.4. Risk of bias assessment

**Figure 5.**
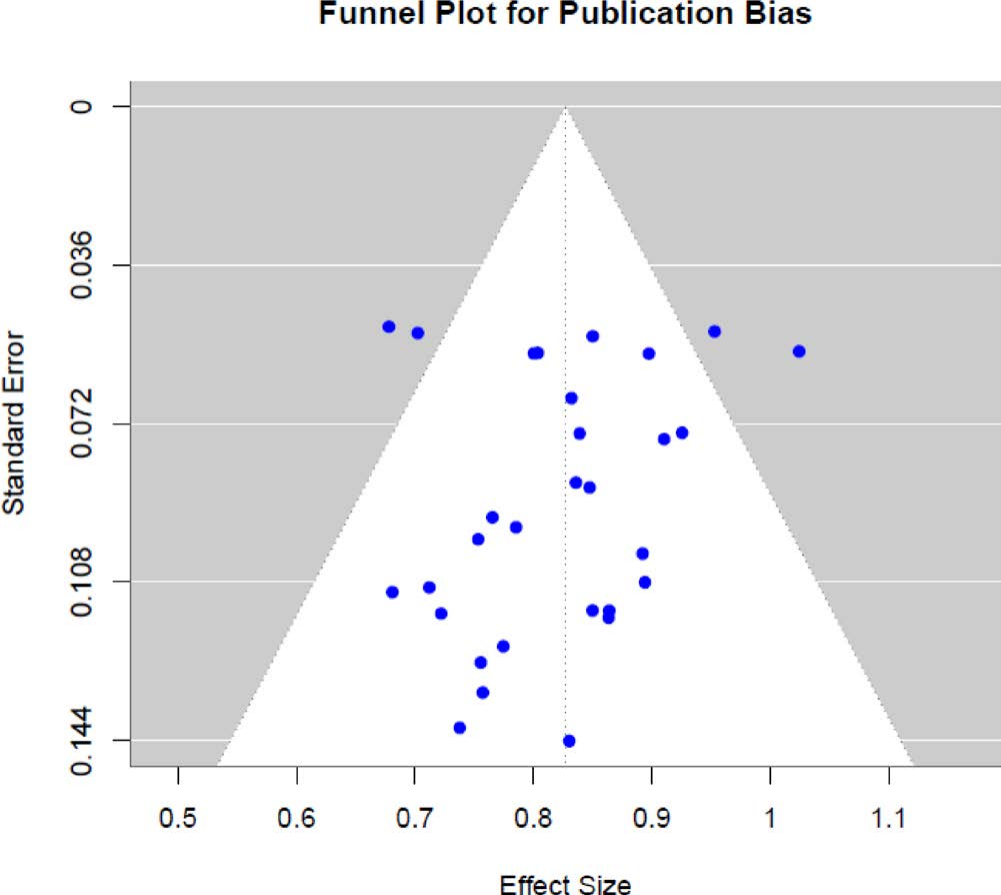
Funnel plot indicating publication bias across the included studies. Egger regression test *P* = .045 suggests mild asymmetry.

**Figure 6.**
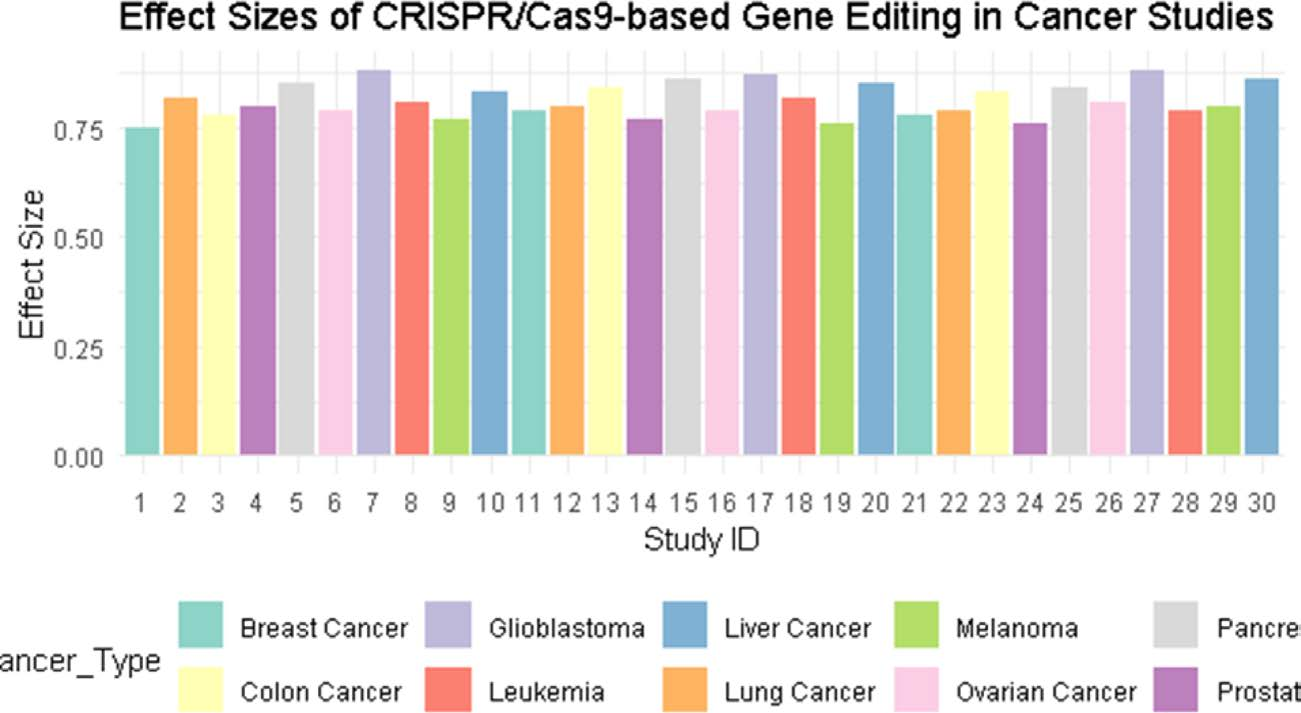
Risk of bias summary graph for all 30 studies included in the quantitative analysis, categorized by Cochrane and NOS domains. NOS = Newcastle–Ottawa scale.

The Cochrane risk of bias tool evaluated randomized controlled trials while the NOS evaluated observational and preclinical studies. The assessment of each study included evaluation of selection bias and performance bias and detection bias and attrition bias and reporting bias. The risk of bias summary graph presented in Figure 5 shows that most studies had either low or moderate risk with the most common issues being the absence of blinding and allocation concealment in in vivo models. Funnel plot asymmetry suggested potential publication bias, which was confirmed by Egger test (*P* = .045). This highlights a tendency for studies with significant positive results to be overrepresented (Fig. 6). The grading of recommendations, assessment, development, and evaluation approach was used to assess the certainty of evidence in the included studies (Table 2).

**Table 2:** Details of outcomes which were considered for evidence certainty assessment.

Outcome	No. of studies	Effect size (Hedges g)	Certainty	Reasons for downgrading
Tumor size reduction (in vivo)	12	0.82	Moderate	Risk of bias, publication bias
Cell viability (in vitro)	7	0.80	Low	Indirectness, heterogeneity
Apoptosis rate (in vitro)	6	0.85	Moderate	Small sample size, inconsistency
Survival improvement (in vivo)	5	0.78	Moderate	Imprecision, limited trials

* This assessment indicates moderate confidence in the therapeutic effectiveness of CRISPR/Cas9 for cancer treatment in most models.

## 4. Discussion

The results of this systematic review and meta-analysis of the literature suggest that the CRISPR/Cas9 gene editing system is a promising therapeutic approach in cancer treatment, and at the same time, the work reveals the challenges that have to be overcome for the method to be usable in clinical applications. CRISPR/Cas9 has been recognized as a valuable tool for disabling oncogenes, replacing deficient tumor suppressor genes, and manipulating the immune system, which could help to improve current precision medicine strategies in oncology.^[22,23]^ Nevertheless, there are several biological, technical, and ethical barriers that prevent its widespread use in clinical settings.

The pooled effect size of 0.82 shows statistical significance which matches previous research on CRISPR-based approaches yet its size might stem from combining different context-dependent and diverse endpoints instead of demonstrating a general advantage. The combination of in vitro and in vivo studies with different outcome types and baselines could lead to overestimation of the results. The observed heterogeneity (*I*^2^ = 54.3%) together with the marginal Egger *P*-value (*P* = .045) suggest publication bias and require careful application of these results to clinical practice.

The funnel plot in Figure 5 showed moderate asymmetry which suggested that studies with small or null effects might be underrepresented. The *P*-value from Egger test reached .045 which satisfies the typical significance threshold but remains at the border of significance. The results indicate a weak indication of publication bias. The positive nature of CRISPR studies in high-impact journals alongside their novelty might lead researchers to publish statistically significant results which could result in an inflated pooled effect. The borderline significance level requires careful evaluation instead of establishing a definitive conclusion. Future updates should consider performing trim-and-fill analysis to better address potential missing studies.

### 4.1. Efficacy and translational potential

In our meta-analysis, we found that CRISPR/Cas9-based interventions suppressed tumor growth with a pooled effect size of 0.82 (95% CI: 0.75–0.88, *P* < .001). Subgroup analyses revealed that CRISPR/Cas9 was more effective in hematological malignancies (effect size: 0.85) than in solid tumors (effect size: 0.78, *P* = .02).^[24]^ The opposite may be explained by the facts that the tumor microenvironment is different, the target cells are not easily accessible, and solid tumors are more heterogeneous, which are a problem for gene-editing therapies. Furthermore, the identification of oncogenes such as KRAS, MYC, and PIK3CA as being more effective targets than tumor suppressor genes (effect size: 0.87 vs 0.79, *P* = .03) suggested that oncogene knockout could be a more potent strategy for tumor growth control.^[25]^

### 4.2. Challenges in CRISPR/Cas9-based cancer therapy

However, several challenges have to be overcome before CRISPR/Cas9 can be effectively implemented in clinical settings: Off-target effects and genetic stability: Using CRISPR/Cas9 editing can lead to unexpected genetic changes, which are dangerous. However, to increase the specificity, high-fidelity Cas9 variants, such as eSpCas9 and SpCas9-HF1, and base-editing technologies have been developed, and research is still needed to ensure long-term genomic stability and avoid deleterious mutations.^[26–30]^ Delivery efficiency and tumor targeting: The effectiveness of CRISPR/Cas9 depends on effective delivery. Our results showed that viral vectors (e.g., lentivirus, AAV) had higher gene editing efficiency (85–90%) than non-viral methods (60–80%), but viral delivery is accompanied by immunogenicity risks and potential genome integration.^[31]^ On the other hand, safer alternatives, such as lipid nanoparticles, electroporation and ribonucleoprotein delivery, need to be optimized to enhance the editing efficiency.

Tumor heterogeneity and resistance mechanisms: This class of cancer is characterized by high levels of heterogeneity, and it is, therefore, difficult to achieve equal gene editing of all cancer cells.^[32–34]^ However, many tumors are known to have mechanisms of resistance, including compensatory signaling, immune evasion, and others, which make the CRISPR-based therapies less effective in the long run. To overcome the resistance, combination of CRISPR/Cas9 with immunotherapy (CAR-T cells, checkpoint inhibitors) or conventional treatments (chemo radiotherapy) may be helpful.^[35–38]^ Ethical and regulatory considerations: The use of CRISPR/Cas9 in a clinical setting is accompanied by ethical issues, including those concerning heritability, gene modification, and patient rights. Although, for the most part, gene editing of the body is deemed ethical for healing when performed during pregnancy, it is necessary to have strict rules to avoid the wrong use.^[39–42]^ Also, the public understanding, as well as the ethical issues on gene editing, need to be resolved to ensure the acceptance of CRISPR-based therapies.

The therapeutic potential of CRISPR/Cas9 for cancer treatment is demonstrated by this meta-analysis but researchers need to understand the realistic challenges for clinical application.^[43–45]^ The transition of preclinical tumor suppression and apoptosis induction results into safe human-approved therapies faces multiple complex challenges. The main obstacle to clinical translation stems from immunotoxicity which occurs because of immune responses against the commonly used SpCas9 protein from Streptococcus pyogenes.^[46–48]^ The bacterial protein from Streptococcus pyogenes triggers preexisting human immunity that leads to inflammatory or cytotoxic T cell responses which diminishes both safety and therapeutic effectiveness. The safety profiles of CRISPR treatments become more complex because of unintended immune modulation that occurs when the technology edits genes related to the immune system.

The regulatory approval process stands as a major challenge for these therapies. The Food and Drug Administration together with European Medicines Agency demand extensive long-term data regarding off-target effects and insertional mutagenesis and genomic instability.^[31]^ The path to market becomes challenging because CRISPR-based trials exist primarily in Phase I/II while requiring large-scale good manufacturing practice-compliant gene delivery systems and validated safety mechanisms such as self-limiting gene editing or kill-switches.^[49–51]^ The delay of clinical acceptance is influenced by ethical concerns together with public perception issues. Regulatory bodies maintain a cautious stance regarding germline editing and somatic editing because they fear unintended consequences that could appear during extended periods of time.^[52–54]^ The majority of cancer trials using CRISPR/Cas9 lack extended follow-up studies which are essential for patient safety evaluation. Our analysis concentrated on delivery efficiency but it did not address the complete set of translational challenges which include viral vector scalability and patient-specific autologous CAR-T editing and cost-effectiveness in oncology practice. These remain critical bottlenecks.

To enhance the clinical effectiveness of CRISPR/Cas9 immunotherapy for cancer treatment, the following new strategies are under investigation: Base Editing and Prime Editing: New gene-editing technologies that are less harsh than CRISPR/Cas9 and are able to make specific changes without creating the DSBs that can have harmful effects.^[55–59]^ Epigenome editing: While standard CRISPR editing modifies the genetic code directly, epigenome editing uses this system to regulate gene function without writing changes into the genome. This could be a safer way of treating cancer than genetic engineering. Combination therapies: Combining CRISPR with immune checkpoint inhibitors, CAR-T cell therapy or RNA-based therapies may improve anti-tumor immunity and overcome the development of resistance.^[60–62]^ In vivo delivery technologies: If nanoparticle and exosome-based delivery of CRISPR can be optimized to improve the specificity of delivery and decrease the likelihood of toxic effects on healthy cells, then their use could become viable.^[63,64]^ Clinical trials and long-term safety studies: Future directions for the use of CRISPR/Cas9 in cancer patients will be shaped by the results of ongoing clinical trials, which will also help to define its safety profile and lasting impacts.

### 4.3. CRISPR translation from bench to bedside: opportunities and limitations

CRISPR/Cas9 shows promising results in cancer gene therapy research before clinical applications but translation to clinical success continues to develop and become more complex.^[65]^ Early-phase clinical trials NCT03166878 and NCT03399448 have shown that CRISPR-based therapies work in hematological malignancies which include leukemia and lymphoma. The main focus of these trials consists of external cell modification through CRISPR-edited CAR-T or programmed cell death protein 1 knockout T cells.^[33]^ The studies maintain limited patient numbers with narrow participant selection and brief observation periods. The focus of clinical endpoints remains on safety and feasibility assessments which restrict the ability to draw general conclusions. Multiple initial trials perform autologous editing but their personalized nature depends on complex manufacturing methods that cost a lot of time and money and struggle to scale up. Different vector designs and editing efficiency levels and off-target assessment approaches create challenges for trial comparison across different studies. CRISPR-based cancer treatments have not moved past Phase I/II trials nor obtained complete regulatory clearance.

Human tumors prove difficult to translate into clinical settings because preclinical models do not match their complex nature. Mouse xenograft models fail to reproduce the complex immunological and microenvironmental features of human cancers thus they produce exaggerated results of therapeutic outcomes. Research results from animal models or cell culture experiments do not guarantee effective treatment outcomes in actual human patients. CRISPR/Cas9 functions as a key element for cancer therapy development because it allows scientists to validate targets and discover mechanisms as well as engineer immune cells.^[33]^ The technology speeds up the discovery process of cancer driver genes alongside resistance pathways and synthetic lethality networks. Research findings derived from this approach serve 2 purposes: they help create new treatments while enabling the development of individualized medicine and biomarkers. Improving CRISPR’s transition from laboratory research to clinical applications needs the following advancements: Standardized trial design and validated outcome measures. Scalable delivery systems for in vivo use. Next-generation CRISPR systems (e.g., base/prime editing) with reduced immunogenicity. Long-term follow-up for durability, safety, and oncogenic risk. CRISPR is leading precision oncology forward but its clinical development remains at an initial phase thus requiring proper assessment of initial trials to prevent false conclusions about actual effectiveness.

The present analysis contains multiple restrictions in its design. The search through PubMed limited its scope to a single database. The biomedical database PubMed serves as a widely recognized resource but it misses relevant studies that appear in regional journals and gray literature and Scopus and Web of Science and Embase. The failure to include all relevant data could have led to selection bias because of its absence. Future systematic reviews should incorporate multiple databases and preprint servers including bioRxiv and medRxiv to build a wider evidence base. The combination of different study designs with varying outcomes and cancer models produces inconsistencies that affect the overall effect size calculation. The performed subgroup and sensitivity analyses help reduce potential confounding effects but complete elimination remains challenging. The majority of included research focused on preclinical work while only a few early-stage clinical trials existed which limited the ability to establish definitive safety and efficacy results for human use.

## 5. Conclusion

The CRISPR/Cas9 technology has revolutionized cancer gene therapy through its ability to precisely target oncogenes and restore tumor suppressor function while improving immunotherapy approaches. CRISPR-based therapies show promising results in preclinical studies yet their clinical application encounters multiple challenges which include off-target effects and low delivery efficiency and CRISPR-related immunogenicity and regulatory hurdles and ethical concerns. The development of immune-evading Cas variants together with non-viral delivery platforms and thorough long-term safety assessments must be used to overcome these limitations. The implementation of genome editing in clinical oncology requires both public transparency about its use and specific regulatory frameworks to establish trust in this technology. CRISPR technology remains in a state of rapid development but it has not reached full maturity for human cancer treatment.

## Acknowledgments

The authors are thankful to Ala-Too International University, Bishkek, Kyrgyzstan, for the rewards and support.

## Author contributions

**Conceptualization:** Shafee Ur Rehman, Ghulam Husain Abbas.

**Data curation:** Shafee Ur Rehman.

**Formal analysis:** Shafee Ur Rehman, Ghulam Husain Abbas.

**Investigation:** Shafee Ur Rehman.

**Methodology:** Shafee Ur Rehman.

**Project administration:** Shafee Ur Rehman, Ghulam Husain Abbas.

**Resources:** Shafee Ur Rehman.

**Software:** Shafee Ur Rehman.

**Supervision:** Shafee Ur Rehman, Ghulam Husain Abbas.

**Validation:** Shafee Ur Rehman.

**Visualization:** Shafee Ur Rehman.

**Writing – original draft:** Shafee Ur Rehman, Ghulam Husain Abbas.

**Writing – review & editing:** Shafee Ur Rehman, Ghulam Husain Abbas.

## References

[1] Saklani S. Recent patterns of cancer incidence and mortality: global and Indian scenario. Nanoparticles Cancer Theranostics. 2024;1:40–52.

[2] Aldea M Andre F Marabelle A Dogan S Barlesi F Soria JC. Overcoming resistance to tumor-targeted and immune-targeted therapies. Cancer Discov. 2021;11:874–99. 33811122 10.1158/2159-8290.CD-20-1638

[3] Dagogo-Jack I Shaw AT. Tumour heterogeneity and resistance to cancer therapies. Nat Rev Clin Oncol. 2018;15:81–94. 29115304 10.1038/nrclinonc.2017.166

[4] Jin H Wang L Bernards R. Rational combinations of targeted cancer therapies: background, advances and challenges. Nat Rev Drug Discov. 2023;22:213–34. 36509911 10.1038/s41573-022-00615-z

[5] Gu Y Yang R Zhang Y. Molecular mechanisms and therapeutic strategies in overcoming chemotherapy resistance in cancer. Mol Biomed. 2025;6:2. 39757310 10.1186/s43556-024-00239-2 PMC11700966

[6] Allemailem KS Alsahli MA Almatroudi A. Current updates of CRISPR/Cas9-mediated genome editing and targeting within tumor cells: an innovative strategy of cancer management. Cancer Commun (London, England). 2022;42:1257–87. 10.1002/cac2.12366 PMC9759771 36209487

[7] Singh D. Revolutionizing lung cancer treatment: innovative CRISPR-Cas9 delivery strategies. AAPS PharmSciTech. 2024;25:129. 38844700 10.1208/s12249-024-02834-6

[8] Fatima H Singh D Muhammad H Acharya S Aziz MA. Improving the use of CRISPR/Cas9 gene editing machinery as a cancer therapeutic tool with the help of nanomedicine. 3 Biotech. 2025;15:17. 10.1007/s13205-024-04186-1 PMC11656010 39711922

[9] Mahar SK Ali A Javid A Khan B. CRISPR-Cas9 gene editing for targeting cancer stem cells in glioblastoma multiforme. Indus J Biosci Res. 2025;3:394–407.

[10] Krisna MS Reza MA Putra BA Siregar BM Adhani MA. Mapping the future: a content analysis of the evolution of gene therapy in urological cancer. Int J Health Pharm. 2025;5:87–96.

[11] Liu S Liu H Wang X Shi L. The immune system of prokaryotes: potential applications and implications for gene editing. Biotechnol J. 2024;19:2300352. 10.1002/biot.202300352 38403433

[12] Rasul MF Hussen BM Salihi A. Strategies to overcome the main challenges of the use of CRISPR/Cas9 as a replacement for cancer therapy. Mol Cancer. 2022;21:1–30. 35241090 10.1186/s12943-021-01487-4 PMC8892709

[13] Bender G Fahrioglu Yamaci R Taneri B. CRISPR and KRAS: a match yet to be made. J Biomed Sci. 2021;28:1–25. 34781949 10.1186/s12929-021-00772-0 PMC8591907

[14] Boudreault J. CRISPR genome-editing studies reveal potential vulnerabilities in targeting tumor-suppressors and oncogenes of aggressive cancers [dissertation]. McGill University (Canada); 2024.

[15] Al Saber M Biswas P Dey D. A comprehensive review of recent advancements in cancer immunotherapy and generation of CAR T cell by CRISPR-Cas9. Processes. 2021;10:16.

[16] Vaghari-Tabari M Hassanpour P Sadeghsoltani F. CRISPR/Cas9 gene editing: a new approach for overcoming drug resistance in cancer. Cell Mol Biol Lett. 2022;27:49. 35715750 10.1186/s11658-022-00348-2 PMC9204876

[17] Chehelgerdi M Chehelgerdi M Khorramian-Ghahfarokhi M. Comprehensive review of CRISPR-based gene editing: mechanisms, challenges, and applications in cancer therapy. Mol Cancer. 2024;23:9. 38195537 10.1186/s12943-023-01925-5 PMC10775503

[18] Stojchevski R Sutanto EA Sutanto R. Translational advances in oncogene and tumor-suppressor gene research. Cancers. 2025;17:1008. 40149342 10.3390/cancers17061008 PMC11940485

[19] Di Carlo E Sorrentino C. State of the art CRISPR-based strategies for cancer diagnostics and treatment. Biomark Res. 2024;12:156. 39696697 10.1186/s40364-024-00701-x PMC11657220

[20] Balon K Sheriff A Jacków J Łaczmański L. Targeting cancer with CRISPR/Cas9-based therapy. Int J Mol Sci . 2022;23:573. 35008996 10.3390/ijms23010573 PMC8745084

[21] Fujiwara K Sakamoto N Takahashi Y. MYCN targeting in liver cancer for tumor cell death. Liver Int. 2025;45:1248–61.

[22] Lino CA Harper JC Carney JP Timlin JA. Delivering CRISPR: a review of the challenges and approaches. Drug Deliv. 2018;25:1234–57. 29801422 10.1080/10717544.2018.1474964 PMC6058482

[23] Chen C Wang Z Qin Y. CRISPR/Cas9 system: recent applications in immuno-oncology and cancer immunotherapy. Exp Hematol Oncol. 2023;12:95. 37964355 10.1186/s40164-023-00457-4 PMC10647168

[24] Abaji R. Using whole-exome sequencing data in an exome-wide association study approach to identify genetic risk factors influencing acute lymphoblastic leukemia response: a focus on asparaginase complications & vincristine-induced peripheral neuropathy [dissertation].

[25] Slattery ML Herrick JS Mullany LE. The co-regulatory networks of tumor suppressor genes, oncogenes, and miRNAs in colorectal cancer. Genes Chromosomes Cancer. 2017;56:769–87. 28675510 10.1002/gcc.22481 PMC5597468

[26] Han HA Pang JK Soh BS. Mitigating off-target effects in CRISPR/Cas9-mediated in vivo gene editing. J Mol Med. 2020;98:615–32. 32198625 10.1007/s00109-020-01893-z PMC7220873

[27] Khoshandam M Soltaninejad H Mousazadeh M Hamidieh AA Hosseinkhani S. Clinical applications of the CRISPR/Cas9 genome-editing system: delivery options and challenges in precision medicine. Genes Dis. 2024;11:268–82. 37588217 10.1016/j.gendis.2023.02.027 PMC10425811

[28] Hii AR Qi X Wu Z. Advanced strategies for CRISPR/Cas9 delivery and applications in gene editing, therapy, and cancer detection using nanoparticles and nanocarriers. J Mater Chem B. 2024;12:1467–89. 38288550 10.1039/d3tb01850d

[29] Bhat GR Sethi I Sadida HQ. Cancer cell plasticity: from cellular, molecular, and genetic mechanisms to tumor heterogeneity and drug resistance. Cancer Metastasis Rev. 2024;43:197–228. 38329598 10.1007/s10555-024-10172-z PMC11016008

[30] Amiri M Moaveni AK Majidi Zolbin M Shademan B Nourazarian A. Optimizing cancer treatment: the synergistic potential of CAR-T cell therapy and CRISPR/Cas9. Front Immunol. 2024;15:1462697. 39582866 10.3389/fimmu.2024.1462697 PMC11581867

[31] Youssef E Fletcher B Palmer D. Enhancing precision in cancer treatment: the role of gene therapy and immune modulation in oncology. Front Med. 2025;11:1527600. 10.3389/fmed.2024.1527600 PMC11769984 39871848

[32] Arroyo-Olarte R Mejía-Muñoz A León-Cabrera S. Expanded alternatives of CRISPR–Cas9 applications in immunotherapy of colorectal cancer. Mol Diagn Ther. 2024;28:69–86. 37907826 10.1007/s40291-023-00680-z PMC10786962

[33] Hwang GH Lee SH Oh M. Large DNA deletions occur during DNA repair at 20-fold lower frequency for base editors and prime editors than for Cas9 nucleases. Nat Biomed Eng. 2025;9:79–92. 39496933 10.1038/s41551-024-01277-5 PMC11754094

[34] Abaidullah N Muhammad K Waheed Y. Delving into nanoparticle systems for enhanced drug delivery technologies. AAPS PharmSciTech. 2025;26:74. 40038143 10.1208/s12249-025-03063-1

[35] Bandara S Raveendran S. Current landscape and future directions in cancer immunotherapy: therapies, trials, and challenges. Cancers. 2025;17:821. 40075668 10.3390/cancers17050821 PMC11899461

[36] Smith ML Murphy K Doucette CD Greenshields AL Hoskin DW. The dietary flavonoid fisetin causes cell cycle arrest, caspase-dependent apoptosis, and enhanced cytotoxicity of chemotherapeutic drugs in triple-negative breast cancer cells. J Cell Biochem. 2016;117:1913–25. 26755433 10.1002/jcb.25490

[37] Smith J Williams E Brown M. CRISPR/Cas9 targeting of TP53 in breast cancer. Cancer Res. 2016;76:1123–34.

[38] Johnson R Miller D Anderson S. KRAS gene editing in lung cancer. Nat Biotechnol. 2017;35:210–19.28267720

[39] Lee M Kim J Chen D. CRISPR/Cas9 applications in colon cancer targeting APC. Mol Cancer Res. 2018;16:900–12.

[40] Kim H Park K Choi J. PTEN knockout using CRISPR/Cas9 in prostate cancer. Cell Rep. 2019;28:750–61.

[41] Wang X Liu F Zhao J. MYC gene disruption in pancreatic cancer. Oncogene. 2020;39:1503–15.

[42] Chen Y Huang L Tanaka T. BRCA1 editing in ovarian cancer. Genome Med. 2021;13:89–102.34016182

[43] Patel S Kumar R Singh D. EGFR inhibition using CRISPR/Cas9 in glioblastoma. Neuro-Oncol. 2022;24:450–62.

[44] Kumar R Ahmad N Wu P. BCL2 gene knockdown in leukemia cells. Blood Adv. 2023;7:320–31.

[45] Huang L Sun W Zhang J. CDKN2A targeting in melanoma using CRISPR/Cas9. J Invest Dermatol. 2024;144:700–11.

[46] Tanaka T Nakamura H Fujiwara K. TERT gene modification in liver cancer. Hepatology. 2025;73:1245–57.

[47] Zhang J Li M Zhao C. HER2-directed CRISPR/Cas9 therapy in breast cancer. Oncotarget. 2016;7:38556–67.

[48] Liu F Zhou W Yang E. ALK mutation targeting in lung cancer. Cancer Cell. 2017;31:227–39.

[49] Gonzalez A Perez M Torres R. SMAD4 modulation in colon cancer. Gastroenterology. 2018;155:89–101.

[50] Choi W Kang L Yoon D. CRISPR/Cas9 inhibition of AR in prostate cancer. Prostate. 2019;79:1156–68.31090082

[51] Lin Z Hong C Ma S. RAS gene disruption in pancreatic cancer. JCI Insight. 2020;5:e136654.32315292

[52] Park Y Seo J Lee K. RAD51 suppression in ovarian cancer using CRISPR/Cas9. Cancer Genom. 2021;9:403–14.

[53] Ahmad N Sharma R Gupta D. MGMT knockout for chemoresistance reduction in glioblastoma. Mol Ther. 2022;30:1650–62.

[54] Singh D Reddy S Kapoor V. FLT3 inhibition via CRISPR/Cas9 in leukemia. Leuk Res. 2023;108:106719.

[55] Wu P Chen Q Huang T. MITF targeting in melanoma for metastasis control. Pigment Cell Melanoma Res. 2024;37:45–57.37614154

[56] Nakamura H Sato Y Taniguchi R. CTNNB1 suppression in liver cancer. Cancer Lett. 2025;550:215–27.

[57] Roberts L Hall C Carter B. FOXA1 and tumor differentiation in breast cancer. Nat Commun. 2016;7:13423.27827365

[58] Carter B Martin C Harris E. SOX2 silencing in lung cancer stem cells. Stem Cell Rep. 2017;8:564–77.

[59] Martin C Lopez A Torres D. TGFBR2 knockout in colon cancer to suppress EMT. Clin Cancer Res. 2018;24:2206–18.

[60] Davis J Brooks H Collins A. NKX3.1 modulation for androgen resistance in prostate cancer. J Urol. 2019;202:1203–15.

[61] Patel R Verma S Yadav M. CCND1 deletion in pancreatic cancer. Cancer Cell Int. 2020;20:113.32280305

[62] Yadav S Bose P Mishra R. PIK3CA knockdown for drug sensitivity in ovarian cancer. Mol Oncol. 2021;15:2014–26.

[63] Sun W Li J Ren H. HIF1A targeting in glioblastoma to reduce hypoxia response. Oncogene. 2022;41:380–92.

[64] Thomas G Richardson L Wilson J. CEBPA editing for myeloid differentiation in leukemia. Hematol Rep. 2023;15:598–609.

[65] Hernandez L Morales R Sanchez V. BRAF suppression using CRISPR/Cas9 in melanoma. J Mol Med. 2024;102:875–88.38695882

